# Supplementary material for: Single-cell transcriptomics reveal the dynamic of haematopoietic stem cell production in the aorta
Source: Nat Commun. 2018 Jun 28;9:2517. doi: 10.1038/s41467-018-04893-3 (PMC6023921; doi:10.1038/s41467-018-04893-3)
Supplement: Supplementary file 1 — Supplementary Information [file 41467_2018_4893_MOESM1_ESM.pdf]

Supplementary Information for:

Single-cell transcriptomics reveal the dynamic of haematopoietic stem cell production in the aorta

Baron *et al.*

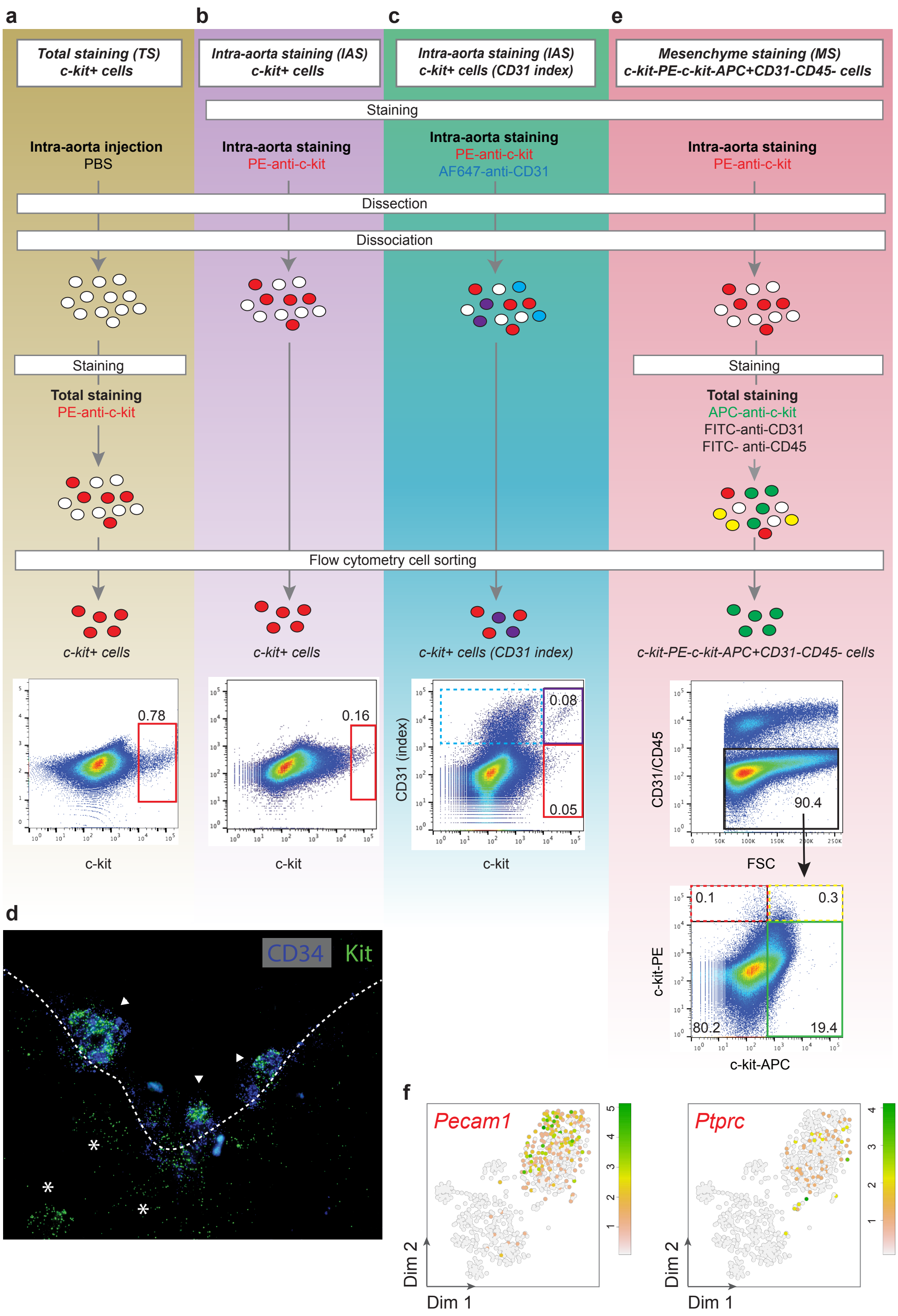
a
b
c
e
Total staining (TS) c-kit+ cells
Intra-aorta staining (IAS) c-kit+ cells
Intra-aorta staining (IAS) c-kit+ cells (CD31 index)
Mesenchyme staining (MS) c-kit-PE-c-kit-APC+CD31-CD45- cells
Staining
Intra-aorta injection
PBS
Intra-aorta staining
PE-anti-c-kit
Intra-aorta staining
PE-anti-c-kit
AF647-anti-CD31
Intra-aorta staining
PE-anti-c-kit
Dissection
Dissociation
Staining
Total staining
PE-anti-c-kit
Staining
Total staining
APC-anti-c-kit
FITC-anti-CD31
FITC- anti-CD45
Flow cytometry cell sorting
c-kit+ cells
c-kit+ cells
c-kit+ cells (CD31 index)
c-kit-PE-c-kit-APC+CD31-CD45- cells
0.78
c-kit
0.16
c-kit
CD31 (index)
0.08
0.05
c-kit
CD31/CD45
90.4
FSC
0.1
0.3
c-kit-PE
80.2
19.4
c-kit-APC
d
CD34
Kit
f
Pecam1
Dim 2
Dim 1
Ptprc
Dim 2
Dim 1

Supplementary Figure 1 (related to Figure 1)**. Antibody staining and cell sorting strategies to isolate IAHC cells from E11 AGMs. (a)** After intra-aortic injection of PBS (to flush the blood out), AGMs were dissected, dissociated and cells were stained with PE anti-c-kit antibodies (procedure referred to as total staining or TS). c-kit^{+} cells were sorted by flow cytometry (red gate in FACS plot) (cells depicted as brown dots in Fig. 1a). **(b)** Cells were stained after intra-aortic injection of PE anti-c-kit antibodies in the aorta (procedure referred to as intra-aortic staining or IAS). AGMs were dissected, dissociated and c-kit^{+} cells were sorted (red gate in FACS plot) (cells depicted as purple dots in Fig. 1a). **(c)** Cells were stained after intra-aorta injection of PE anti-c-kit and AF647 anti-CD31 antibodies. AGMs were then dissected, dissociated and c-kit^{+} cells were sorted with CD31 sorting index (red and purple gates in FACS plot) (c-kit^{+}CD31^{+} cells and c-kit^{+}CD31^{-} cells depicted as green and blue dots in Fig. 1a, respectively). **(d)** Single-molecule fluorescent *in situ* hybridization (smFISH) on an E11 embryo cryosection with probes against *CD34* (blue dots) and *Kit* (green dots). Cells expressing *Kit* transcripts are present in IAHCs (arrow heads) and in the mesenchyme underneath the aortic endothelium (asterisks). **(e)** Cells were stained after intra-aorta injection of PE anti-c-kit antibodies. AGMs were dissected, dissociated and cells were stained with APC anti-c-kit, FITC anti-CD31 and FITC anti-CD45 antibodies. PE-c-kit^{-}APC-c-kit^{+}CD31^{-}CD45^{-} cells were sorted (green gate in FACS plot) (cells depicted as pink dots in Fig. 1a). Percentages of viable cells are indicated in the gates. **(f)** Expression of *Pecam1* and *Ptprc* marker genes projected on t-SNE maps. Dim, dimension.

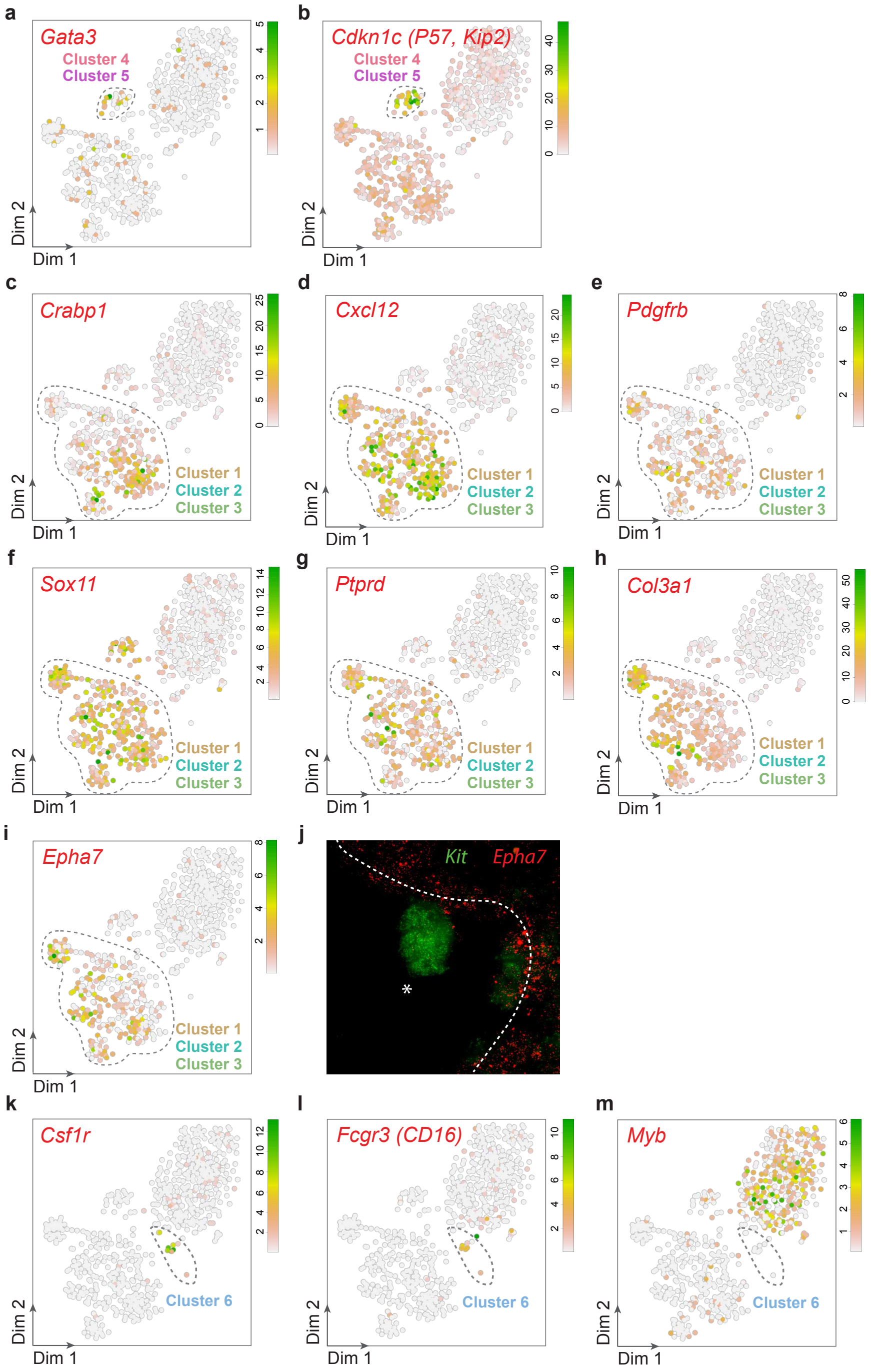

Supplementary Figure 2 (related to Figure 1)**. Marker gene expression reveals the presence of contaminating non-IAHC cells among the sorted IAHC cells. (a-i; k-m)** The transcript count of selected marker genes is projected on t-SNE maps. Shown are maps for sub-aortic patches markers *Gata3* and *Cdkn1c* **(a, b)**, for mesenchymal marker genes *Crabp1* **(c)**, *Cxcl12* **(d)**, *Pdgfrb* **(e)**, *Sox11* **(f)**, *Ptprd* **(g)**, *Col3a1* **(h)**, *Epha7* **(i)**, and for erythro-myeloid progenitors *Csf1r* **(k)**, *Fcgr3* **(l)** and *Myb* **(m)**. The expression of the above mentioned transcripts identify few cells in RaceID clusters 4 and 5, and all cells in RaceID clusters 1, 2 and 3 in Fig. 1b. Dim, dimension. **(j)** smFISH on an E11 embryo cryosection with probes against *Kit* (green dots) and *Epha7* (red dots). Cells in IAHC (asterisk) expressed *Kit* but not *Epha7* while cells from the underlying mesenchyme expressed *Epha7*.

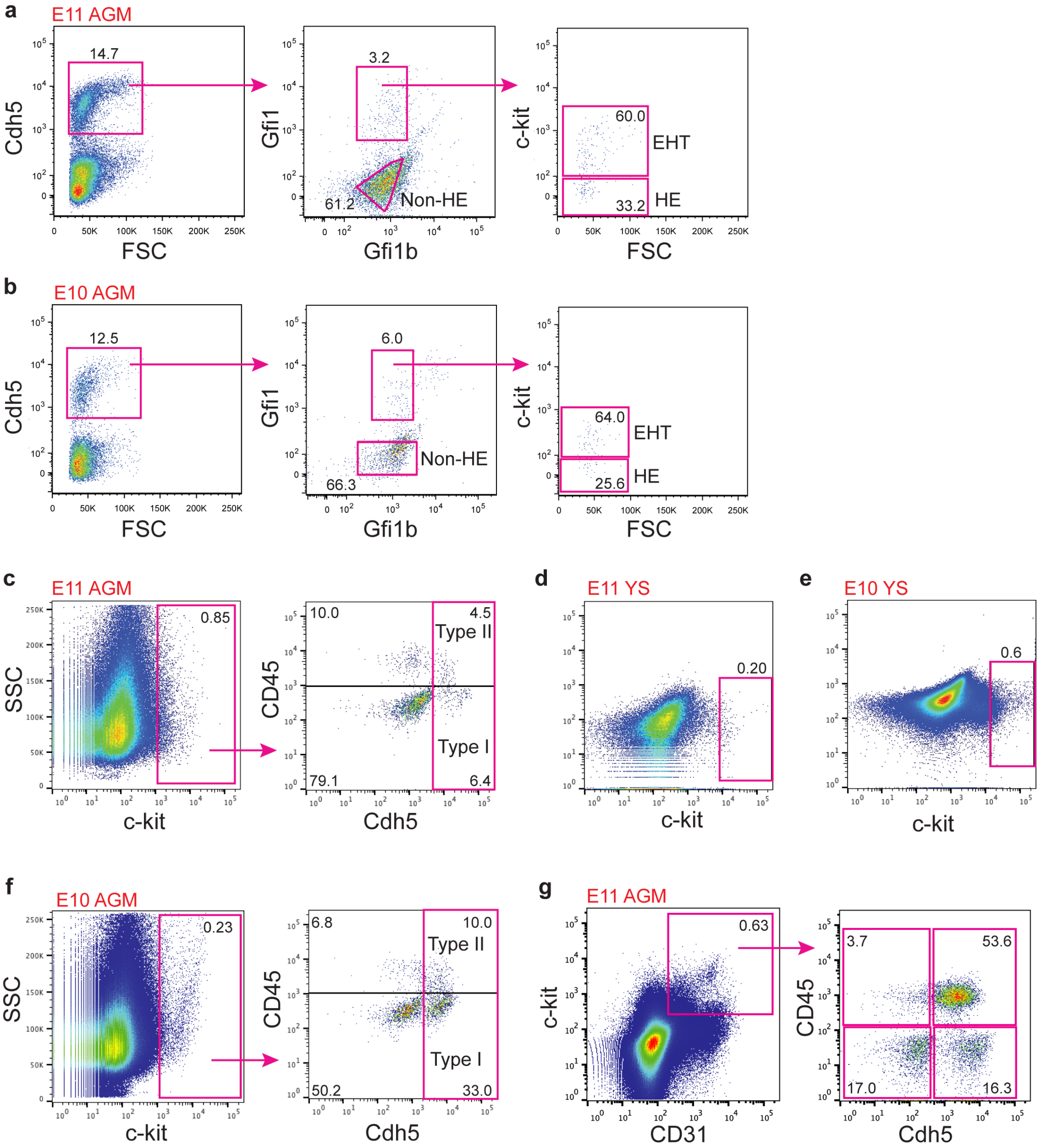

Supplementary Figure 3 (related to Figures 2 and 3)**. Different aortic cell populations sorted from E10 and E11 AGMs and YSs. (a-g)** Examples of cell sorting and gating strategies. **(a)** Cdh5^{+}Gfi1^{-}Gfi1b^{-}c-kit^{-} non haemogenic endothelial (HE) cells, Cdh5^{+}Gfi1^{+}Gfi1b^{-}c-kit^{-} HE cells, and Cdh5^{+}Gfi1^{+}Gfi1b^{-}c-kit^{+} cells undergoing endothelial to haematopoietic transition (EHT) sorted from E11 AGMs for scRNA-seq analysis. **(b)** Non-HE cells, HE cells and EHT cells sorted from E10 AGMs for scRNA-seq analysis. **(c)** Cdh5^{+}c-kit^{+} cells sorted with CD45 index (CD45^{-}, type I pre-HSCs; CD45^{+}, type II pre-HSCs) from E11 flushed AGMs for scRNA-seq analysis. **(d, e)** c-kit^{+} HSPCs sorted from E11 **(d)** or E10 **(e)** YSs for scRNA-seq analysis. **(f)** Cdh5^{+}c-kit^{+} cells sorted with CD45 index (CD45^{-}, type I pre-HSCs; CD45^{+}, type II pre-HSCs) from E10 flushed AGMs for scRNA-seq analysis. **(g)** Cells sorted based on c-kit, CD31, Cdh5 and CD45 differential expression from E11 flushed AGMs for *in vitro* clonogenic assays.

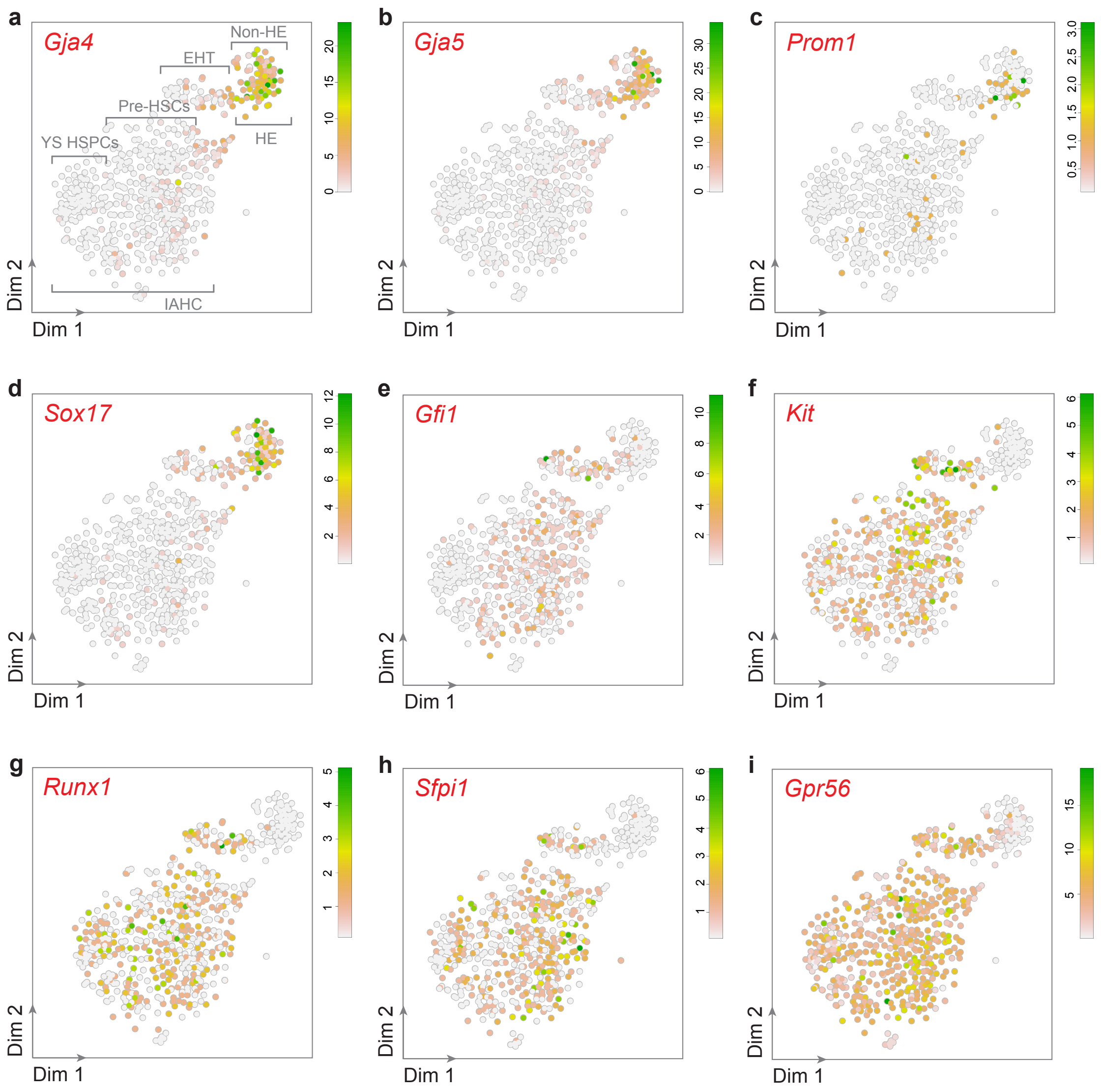

Supplementary Figure 4 (related to Figure 2)**. Evolution of selected marker genes during EHT and IAHC formation at E11. (a-i)** The transcript count of selected marker genes is projected on t-SNE maps. Shown are maps for *Gja4* **(a)**, *Gja5* **(b)**, *Prom1* **(c)**, *Sox17* **(d)**, *Gfi1* **(e)**, *Kit* **(f)**, *Runx1* **(g)**, *Sfpi1* **(h)** and *Gpr56* **(i)**.

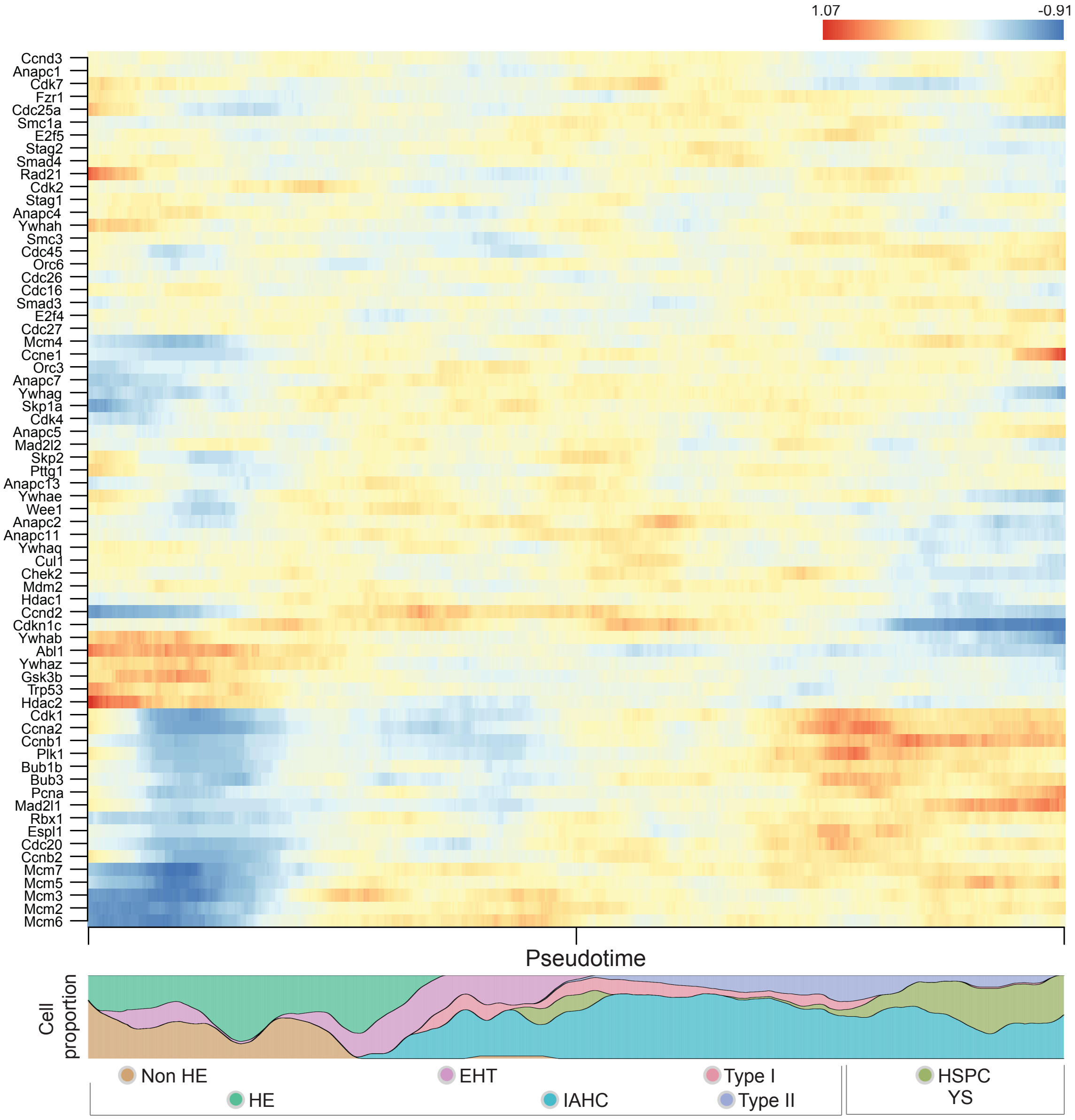

Supplementary Figure 5 (related to Figure 2)**. Dynamic and fluctuant expression of cell cycle related genes at E11, according to pseudotime.** Heatmap depicting on the y-axis the genes related to the KEGG cell cycle pathway and on the x-axis the E11 single cells ordered along the pseudotime axis.

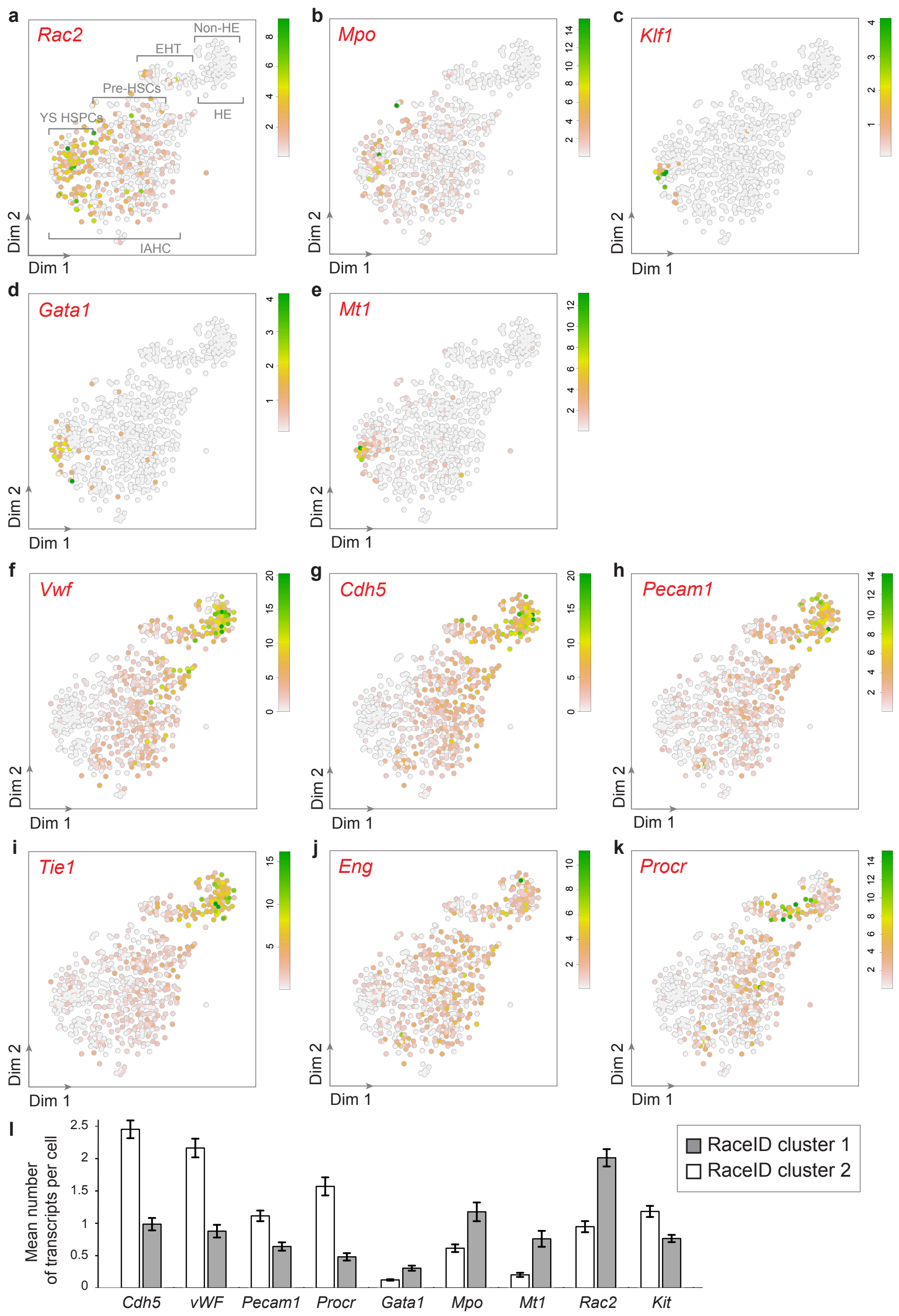

Supplementary Figure 6 (related to Figure 2)**. Opposing gradients of endothelial and haematopoietic transcripts identify committed haematopoietic progenitors and pre-HSCs as the two cell populations composing IAHCs. (a-k)** The transcript count of selected marker genes is projected on t-SNE maps. **(a-e)** Shown are maps for *Rac2* **(a)**, *Mpo* **(b)**, *Klf1* **(c)**, *Gata1* **(d)** and *Mt1* **(e)** (that identify committed haematopoietic progenitors in RaceID cluster 1 in Fig. 2d). **(f-k)** Shown are maps for *Vwf* **(f)**, *Cdh5* **(g)**, *Pecam1* **(h)**, *Tie1* **(i)**, *Eng* **(j)** and *Procr* **(k)** (that identify pre-HSCs in RaceID clusters 2 in Fig. 2d). **(l)** Histogram representing the average numbers of transcripts per cell in RaceID clusters 1 and 2 for the designated genes. Data are represented as mean ± SEM.

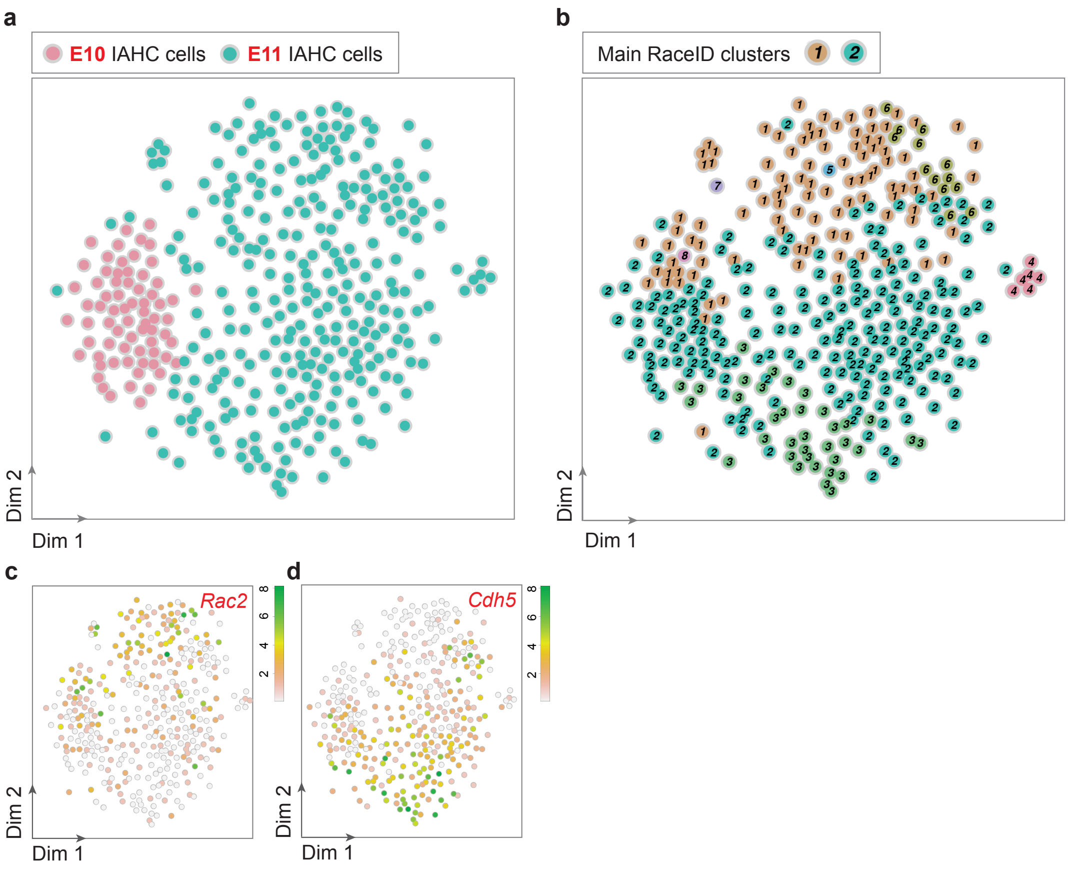

Supplementary Figure 7 (related to Figures 2 and 3)**. Both E10 and E11 IAHCs contain committed progenitors and pre-HSCs. (a)** t-SNE map displaying single IAHC cells isolated from E10 (pink dots) and E11 (green dots) embryo AGMs. **(b)** t-SNE plot displaying single cells from **(a)** in clusters identified by RaceID. Different numbers and colours highlight different RaceID clusters. Cluster 1 contained IAHC progenitors, while cluster 2 contained pre-HSCs. **(c,d)** Distribution of selected marker gene expression in t-SNE maps. *Rac2* expression is restricted to cluster 1 **(c)** while *Cdh5* expression is restricted to cluster 2 **(d)**.

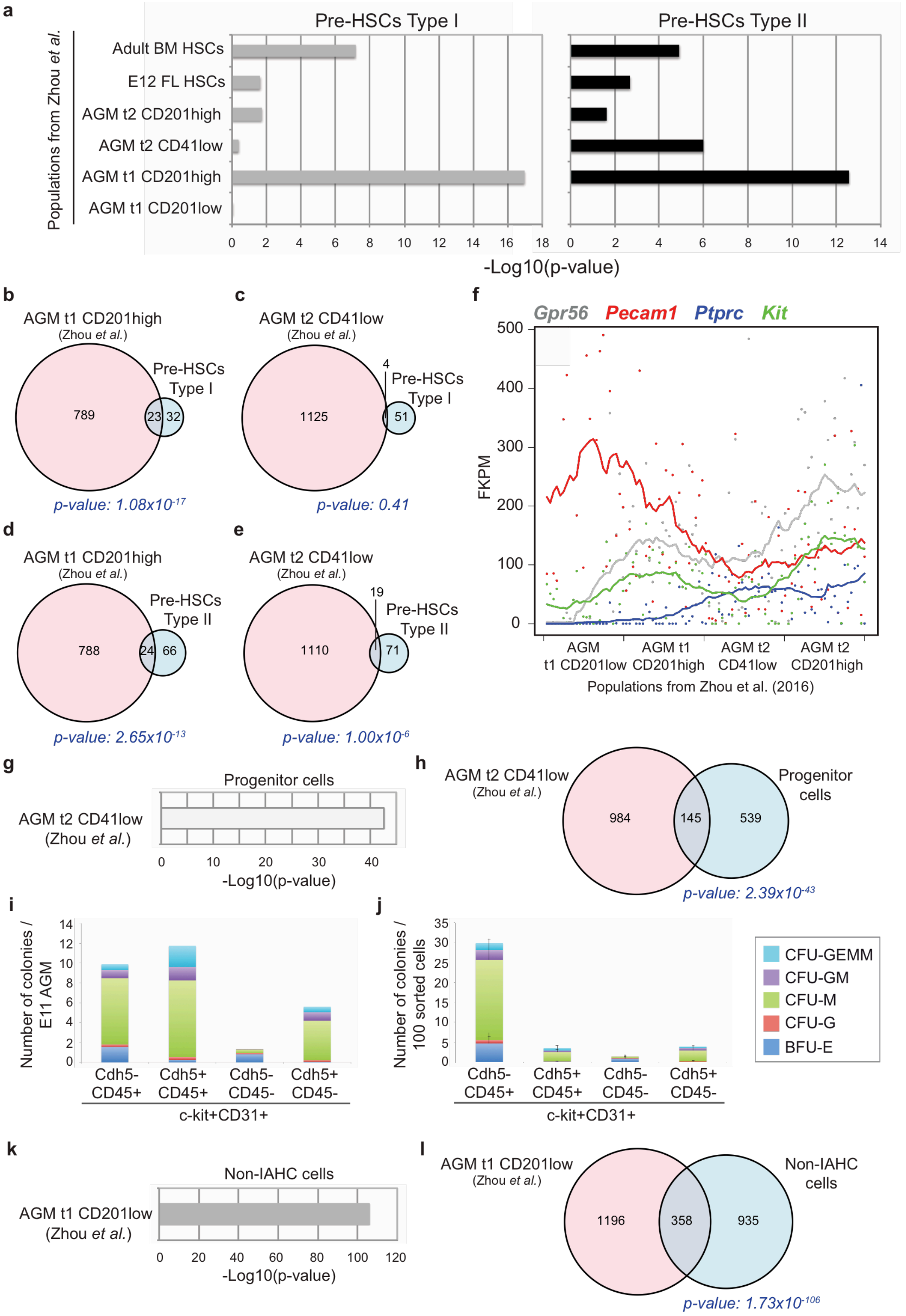

a
Pre-HSCs Type I
Pre-HSCs Type II
Populations from Zhou et al.
Adult BM HSCs
E12 FL HSCs
AGM t2 CD201high
AGM t2 CD41low
AGM t1 CD201high
AGM t1 CD201low
-Log10(p-value)
b
AGM t1 CD201high (Zhou et al.)
Pre-HSCs Type I
789
23
32
p-value: 1.08x10-17
c
AGM t2 CD41low (Zhou et al.)
Pre-HSCs Type I
1125
4
51
p-value: 0.41
d
AGM t1 CD201high (Zhou et al.)
Pre-HSCs Type II
788
24
66
p-value: 2.65x10-13
e
AGM t2 CD41low (Zhou et al.)
Pre-HSCs Type II
1110
19
71
p-value: 1.00x10-6
f
Gpr56 Pecam1 Ptprc Kit
FKPM
AGM t1 CD201low
AGM t1 CD201high
AGM t2 CD41low
AGM t2 CD201high
Populations from Zhou et al. (2016)
g
Progenitor cells
AGM t2 CD41low (Zhou et al.)
-Log10(p-value)
h
AGM t2 CD41low (Zhou et al.)
Progenitor cells
984
145
539
p-value: 2.39x10-43
i
Number of colonies / E11 AGM
Cdh5- CD45+
Cdh5+ CD45+
Cdh5- CD45-
Cdh5+ CD45-
c-kit+CD31+
j
Number of colonies / 100 sorted cells
Cdh5- CD45+
Cdh5+ CD45+
Cdh5- CD45-
Cdh5+ CD45-
c-kit+CD31+
CFU-GEMM
CFU-GM
CFU-M
CFU-G
BFU-E
k
Non-IAHC cells
AGM t1 CD201low (Zhou et al.)
-Log10(p-value)
l
AGM t1 CD201low (Zhou et al.)
Non-IAHC cells
1196
358
935
p-value: 1.73x10-106

Supplementary Figure 8 (related to Figure 2). **Confirmation of pre-HSC and committed progenitor identities in E11 IAHCs. (a, g, k)** The genes differentially expressed between each E11 AGM population from Zhou *et al.*[29] and the genes differentially expressed between each of our E11 AGM populations (Fig.1a and 2a) were compared. The p-values representing the significance of the overlap between the Zhou populations and the pre-HSCs type I and type II are depicted as column bars (-Log10(p-value)) in **(a)**. **(b-e)** Venn diagrams showing shared and distinct genes between **(b)** AGM t1 $CD201^{high}$ and pre-HSCs type I, **(c)** AGM t2 $CD41^{low}$ and pre-HSCs type I, **(d)** AGM t1 $CD201^{high}$ and pre-HSCs type II, **(e)** AGM t2 $CD41^{low}$ and pre-HSCs type II. **(f)** Fragments per kilobase of exon per million reads mapped (FPKM) values for *Gpr56*, *Pecam1*, *Ptprc* and *Kit* in each E11 AGM population from Zhou *et al.*[29]. **(g)** The p-values representing the significance of the overlap between the Zhou populations and the progenitor cells are depicted as column bars (-Log10(p-value)). **(h)** Venn diagrams showing shared and distinct genes between AGM t2 $CD41^{low}$ and progenitor cells. **(i)** *In vitro* clonogenic analyses of E11 AGM cells sorted based on c-kit, CD31, Cdh5 and CD45 differential expression. Bars, number of colonies/E11 AGM embryo equivalent for each indicated sorted population ± SD. n=6 independent experiments. CFU-GEMM: CFU-Granulocyte–Erythroid–Macrophage–Megakaryocyte; CFU-GM: CFU- Granulocyte–Macrophage; CFU-M: CFU-Macrophage; CFU-G: CFU-Granulocyte; BFU-E: Burst-Forming Unit-Erythroid. **(j)** Clonogenic data shown in **(i)** represented in number of colonies/100 sorted cells (for each population indicated on the x-axis). **(k)** The p-values representing the significance of the overlap between the Zhou populations and the non-IAHC cells are depicted as column bars (-Log10(p-value)). **(l)** Venn diagrams showing shared and distinct genes between AGM t1 $CD201^{low}$ and non-IAHC cells.

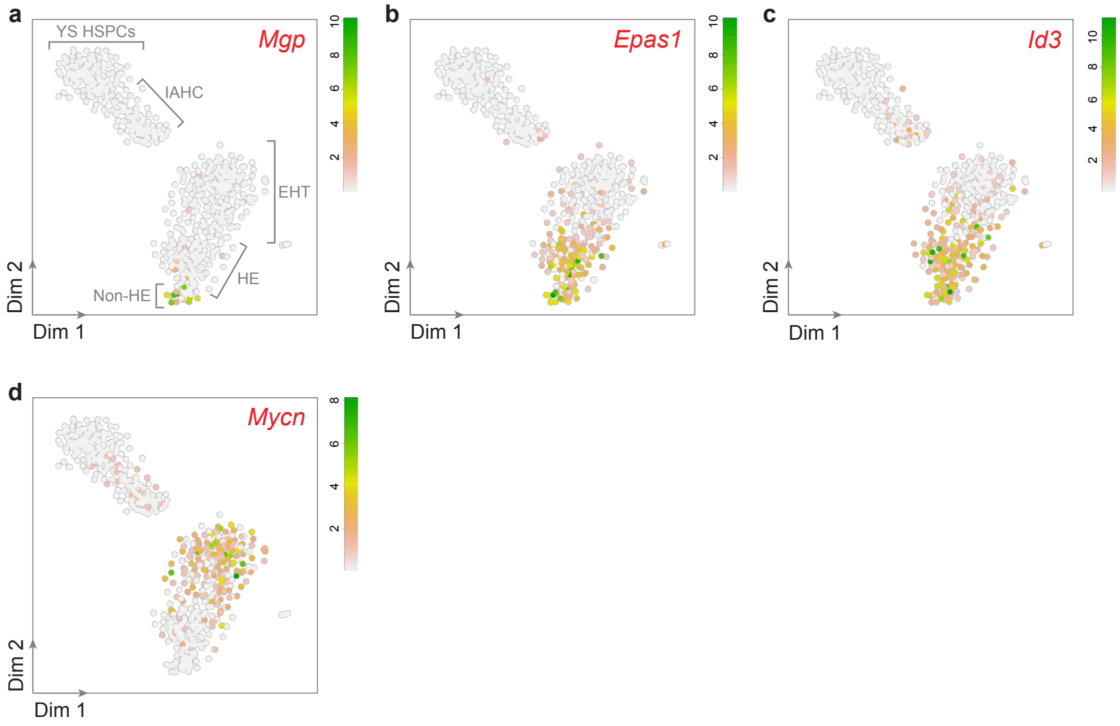

Supplementary Figure 9 (related to Figure 3)**. Evolution of selected marker genes during EHT at E10. (a-d)** The transcript count of selected marker genes is projected on t-SNE maps. Shown are maps for *Mgp* **(a)**, *Epas1* **(b)**, *Id3* **(c)** and *Mycn* **(d)**.

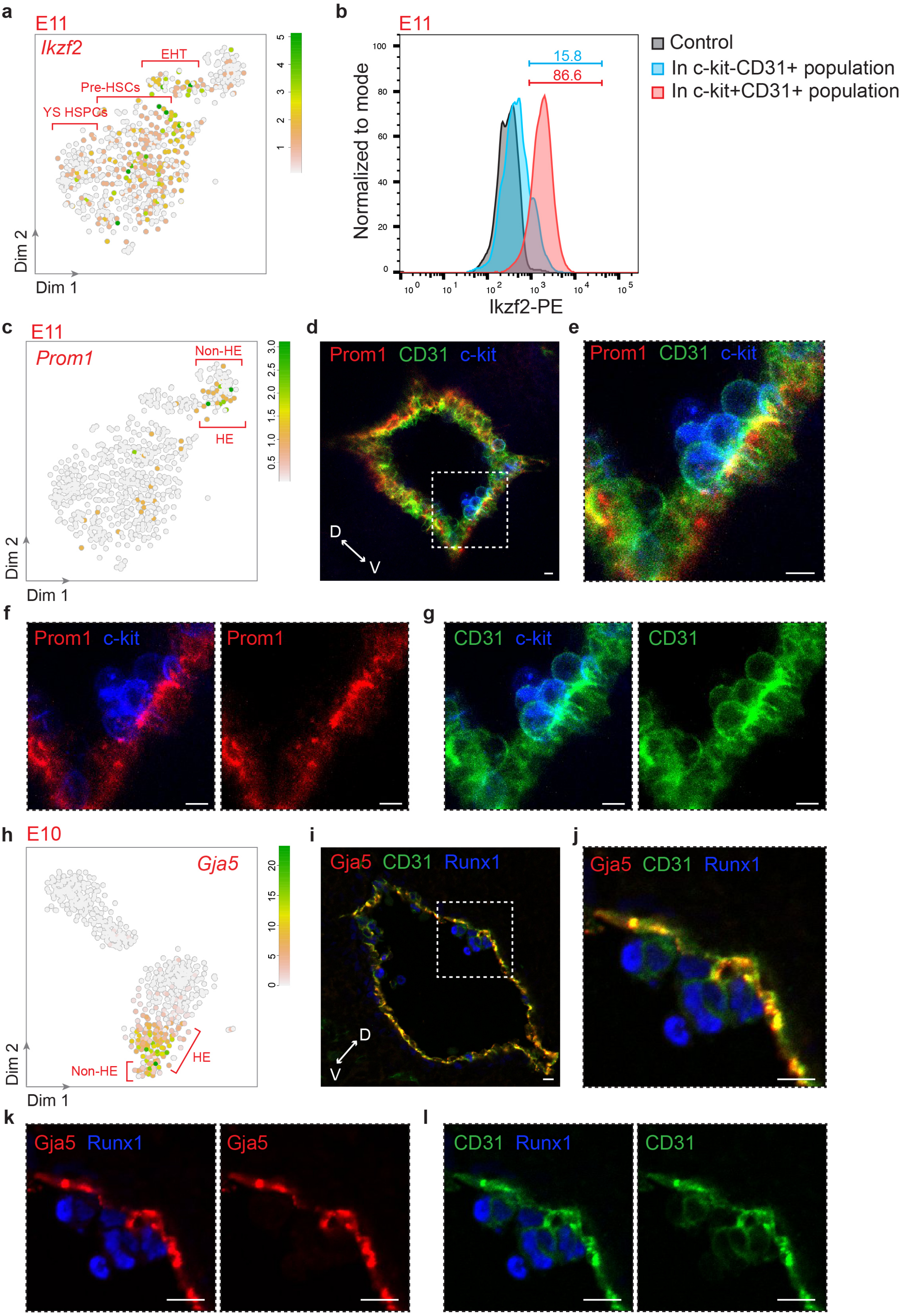
a
E11
Ikzf2
EHT
Pre-HSCs
YS HSPCs
Dim 2
Dim 1
b
E11
Normalized to mode
Ikzf2-PE
Control
In c-kit-CD31+ population
In c-kit+CD31+ population
15.8
86.6
c
E11
Prom1
Non-HE
HE
Dim 2
Dim 1
d
Prom1 CD31 c-kit
D
V
e
Prom1 CD31 c-kit
f
Prom1 c-kit
Prom1
g
CD31 c-kit
CD31
h
E10
Gja5
HE
Non-HE
Dim 2
Dim 1
i
Gja5 CD31 Runx1
D
V
j
Gja5 CD31 Runx1
k
Gja5 Runx1
Gja5
l
CD31 Runx1
CD31

Supplementary Figure 10 (related to Figures 2 and 3)**. Correlation between protein expression patterns and scRNA-seq data for IKZF2, PROM1 and GJA5. (a, c, h)** Distribution of selected marker gene expression in t-SNE maps. *Ikzf2* expression is mainly in IAHC cells **(a)** while *Prom1* **(c)** and *Gja5* **(h)** expression is restricted to endothelial cells. **(b)** Flow cytometry analysis shows IKZF2 expression in c-kit$^-$CD31$^+$ endothelial cells (blue) and c-kit$^+$CD31$^+$ IAHC cells (red) represented in histogram. Control: grey. Lines indicate the percentages of IKZF2$^+$ cells in each live population. **(d-g)** Immunostaining on an E11 live thick embryo slice with antibodies against c-kit (blue), CD31 (green) and PROM1 (red). c-kit$^+$CD31$^+$ IAHC cells do not express Prom1 while c-kit$^-$CD31$^+$ endothelial cells do express Prom1. **(e-g)** Dashed white box in **(d)** is enlarged in **(e-g)**. **(i-l)** Immunostaining on an E10 embryo cryosection with antibodies against RUNX1 (blue), CD31 (green) and GJA5 (red). RUNX1$^+$CD31$^+$ IAHC cells do not express GJA5 while RUNX1$^-$CD31$^+$ endothelial cells do express GJA5. Dashed white box in **(i)** is enlarged in **(j-l)**. Scale bars, 10 μm. V: ventral side of the embryo. D: dorsal side of the embryo.

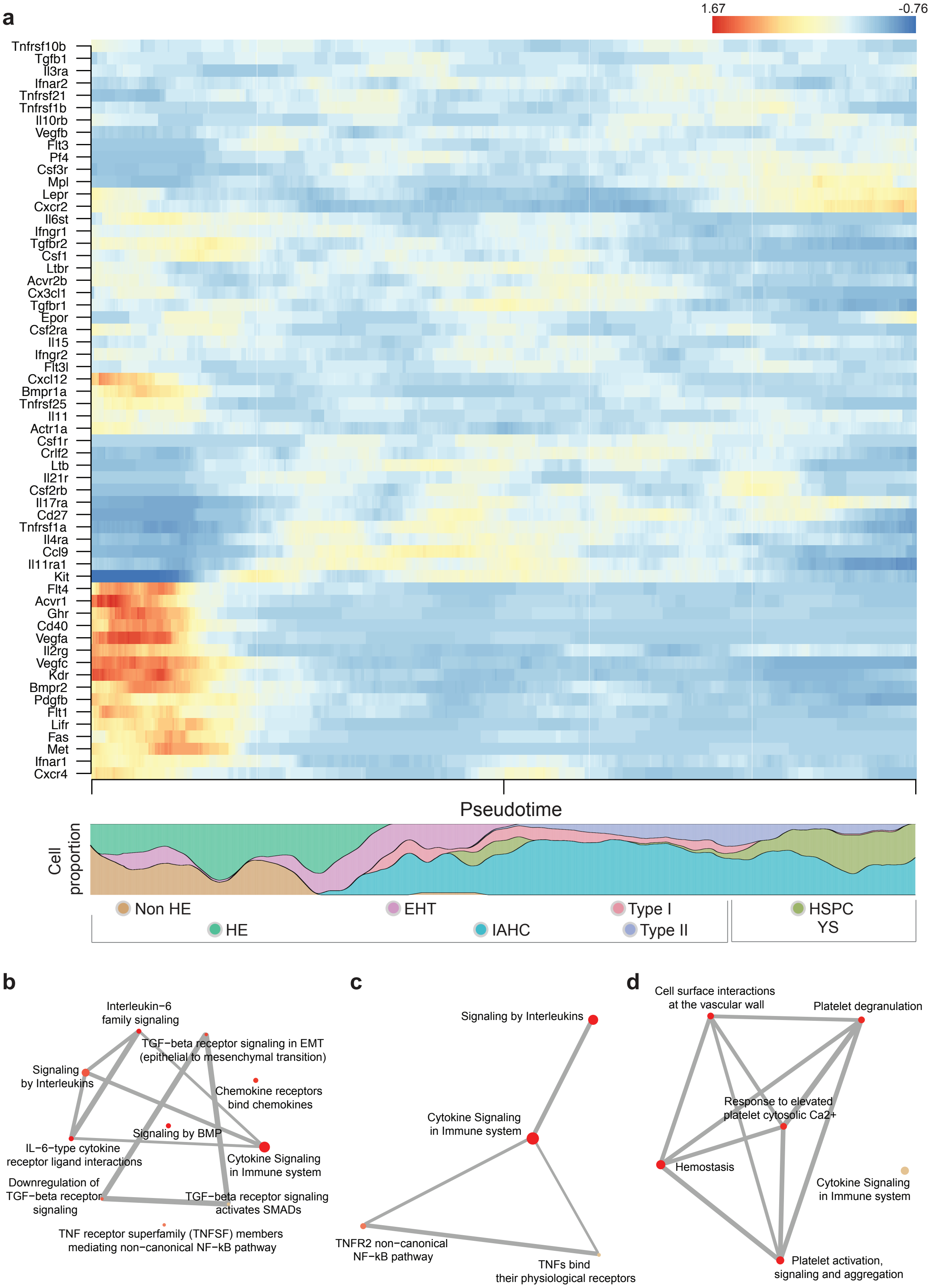
a
1.67
-0.76
Tnfrsf10b
Tgfb1
Il3ra
Ifnar2
Tnfrsf21
Tnfrsf1b
Il10rb
Vegfb
Flt3
Pf4
Csf3r
Mpl
Lepr
Cxcr2
Il6st
Ifngr1
Tgfbr2
Csf1
Ltbr
Acvr2b
Cx3cl1
Tgfbr1
Epor
Csf2ra
Il15
Ifngr2
Flt3l
Cxcl12
Bmpr1a
Tnfrsf25
Il11
Actr1a
Csf1r
Crlf2
Ltb
Il21r
Csf2rb
Il17ra
Cd27
Tnfrsf1a
Il4ra
Ccl9
Il11ra1
Kit
Flt4
Acvr1
Ghr
Cd40
Vegfa
Il2rg
Vegfc
Kdr
Bmpr2
Pdgfb
Flt1
Lifr
Fas
Met
Ifnar1
Cxcr4
Pseudotime
Cell proportion
Non HE
HE
EHT
IAHC
Type I
Type II
HSPC
YS
b
Interleukin−6 family signaling
TGF−beta receptor signaling in EMT (epithelial to mesenchymal transition)
Signaling by Interleukins
Chemokine receptors bind chemokines
Signaling by BMP
IL−6−type cytokine receptor ligand interactions
Cytokine Signaling in Immune system
Downregulation of TGF−beta receptor signaling
TGF−beta receptor signaling activates SMADs
TNF receptor superfamily (TNFSF) members mediating non−canonical NF−kB pathway
c
Signaling by Interleukins
Cytokine Signaling in Immune system
TNFR2 non−canonical NF−kB pathway
TNFs bind their physiological receptors
d
Cell surface interactions at the vascular wall
Platelet degranulation
Response to elevated platelet cytosolic Ca2+
Hemostasis
Cytokine Signaling in Immune system
Platelet activation, signaling and aggregation

Supplementary Figure 11 (related to Figure 2). **Dynamic and fluctuant expression of cytokine/growth factor related genes at E11, according to pseudotime. (a)** Heatmap depicting on the y-axis the genes present in each pathway according to KEGG cytokine pathway and on the x-axis the E11 single cells ordered along the pseudotime axis. **(b-d)** KEGG pathway analysis on genes related to the cytokine pathways (shown in **(a)**), in **(b)** endothelial cells (non HE, HE), **(c)** EHT and IAHC cells (pre-HSCs type I and II), and **(d)** committed progenitors.

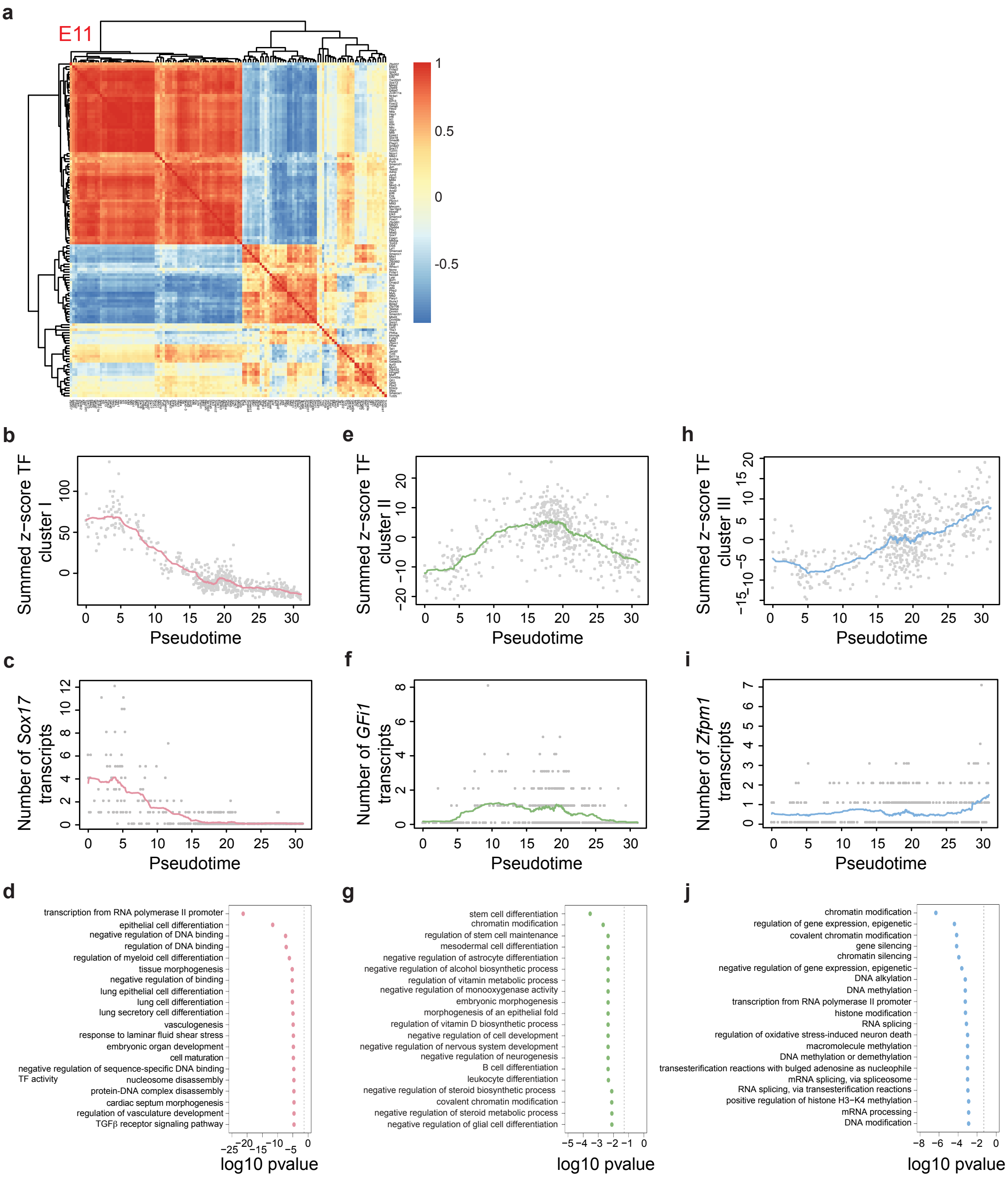

Supplementary Figure 12 (related to Figures 2 and 4)**. Transcription factor expression during EHT and IAHC formation at E11. (a)** Heatmap depicting Euclidean distances between the 127 transcription factors (TFs) obtained in our set of E11 data. **(b, e, h**) Mean z-score of TFs in heatmap clusters I **(b)**, II **(c)** and III **(d)** according to Monocle pseudotime. **(c, f, i)** Mean number of *Sox17* **(c)**, *Gfi1* **(f)** and *Zfpm1* **(i)** transcripts per cell according to pseudotime. Grey dots: single cells. **(d, g, j)** Major biological process GO terms enriched in heatmap clusters I **(d)**, II **(g)** and III **(j)**.

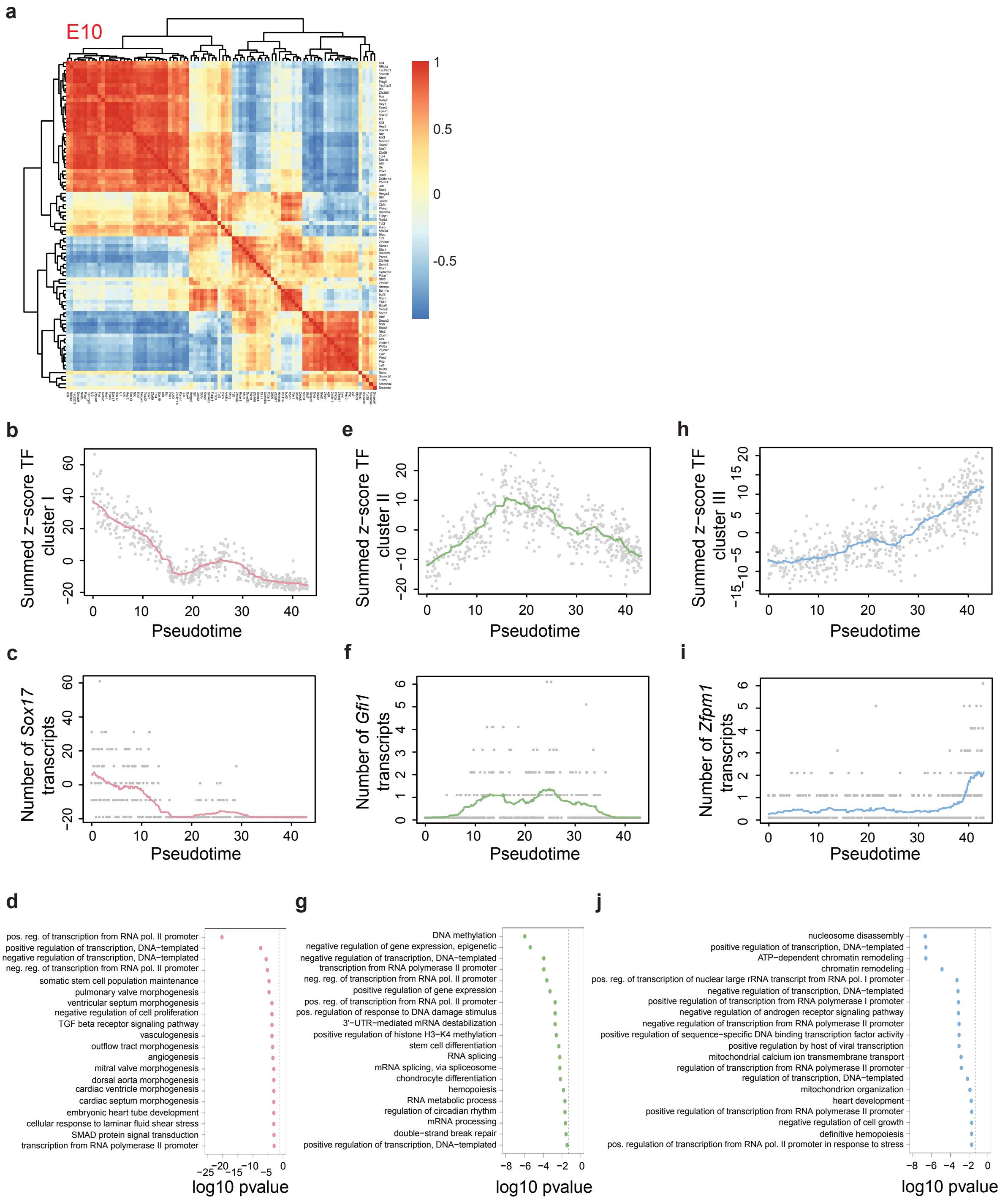

Supplementary Figure 13 (related to Figures 3 and 4)**. Transcription factor expression during EHT and IAHC formation at E10. (a)** Heatmap depicting Euclidean distances between the 88 transcription factors (TFs) obtained in our set of E10 data. **(b, e, h**) Mean z-score of TFs in heatmap clusters I **(b)**, II **(c)** and III **(d)** according to Monocle pseudotime. **(c, f, i)** Mean number of *Sox17* **(c)**, *Gfi1* **(f)** and *Zfpm1* **(i)** transcripts per cell according to pseudotime. Grey dots: single cells. **(d, g, j)** Major biological process GO terms enriched in heatmap clusters I **(d)**, II **(g)** and III **(j)**.

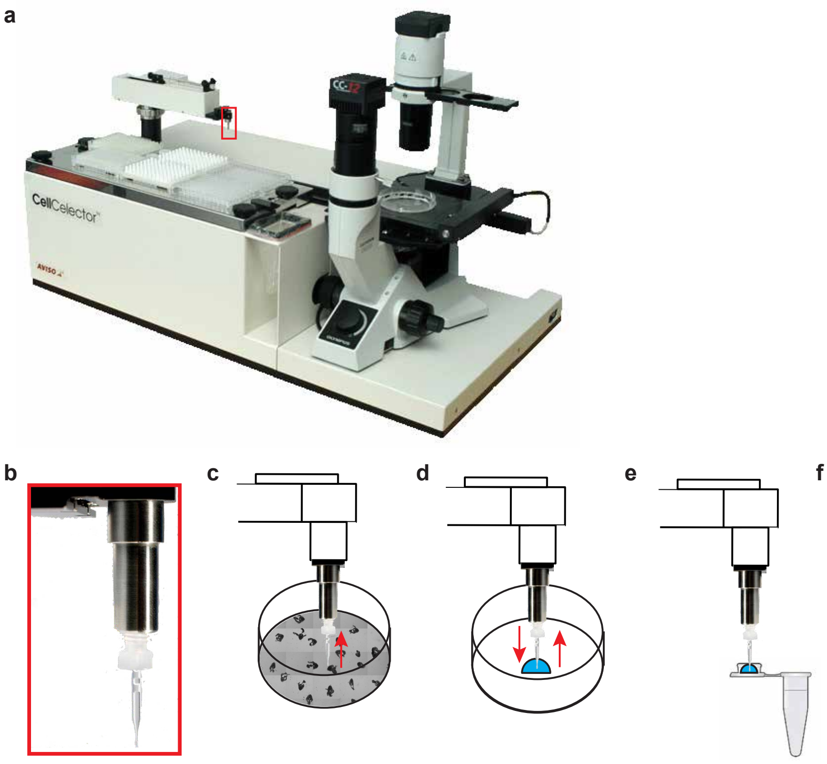

f

	Early E10	E11
Dorsal	10	15
Ventral	25	23

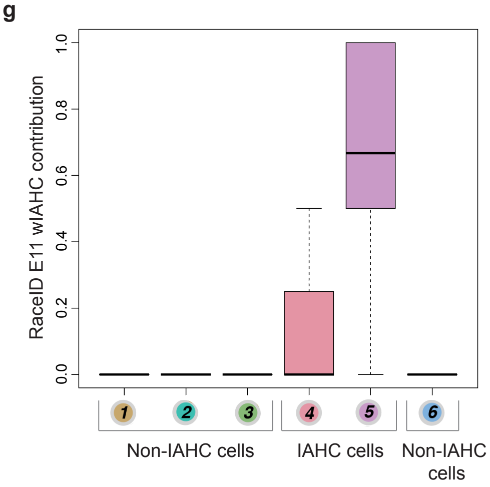

Supplementary Figure 14 (related to Figures 1, 5 and 6)**. Mechanical pick-up of pure wIAHCs in embryo aorta thick slices. (a)** Automated cell picker CellCellector^{TM}. **(b)** Close-up of the harvesting module composed of a glass capillary connected to a syringe, associated with the CellCellector^{TM} robotic arm (red box shown in **(a)**). **(c-e)** Steps of single wIAHC pick-up (see *Methods* for details). **(c)** The first pick-up allowed harvesting of single wIAHCs from the aorta of embryo slides immobilized in agarose in a 6-well plate. **(d)** wIAHCs were deposited (left arrow) and picked-up a second time (right arrow) to eliminate the presence of non-IAHC cells. **(e)** Pure single wIAHCs were collected in individual cap tubes. **(f)** Number of wIAHCs mechanically picked-up in the dorsal and ventral side of the aorta from E10 and E11 embryo slices. **(g)** Box plots of the RaceID cluster contribution to mechanically picked-up E11 wIAHCs. All wIAHCs were only composed of IAHC cells (identified by RaceID clusters 4 and 5) and no contaminating non-IAHC cells (identified by RaceID clusters 1, 2, 3 and 6). Colour-coded boxes are related to RaceID analysis shown in Fig. 1b.
