# Supplementary material for: Single-cell transcriptomics reveal the dynamic of haematopoietic stem cell production in the aorta
Source: Nat Commun. 2018 Jun 28;9:2517. doi: 10.1038/s41467-018-04893-3 (PMC6023921; doi:10.1038/s41467-018-04893-3)
Supplement: Supplementary file 3 — Description of Additional Supplementary Files [file 41467_2018_4893_MOESM3_ESM.pdf]

Description of Additional Supplementary Files

File Name: Supplementary Data 1
Description: List of genes differentially expressed between type I and II pre-HSCs at E11 (Fig. 2e).

File Name: Supplementary Data 2
Description: List of genes differentially expressed between HE cells and EHT cells at E11 (Fig. 3e, left panel).

File Name: Supplementary Data 3
Description: List of genes differentially expressed between HE cells and EHT cells at E10 (Fig. 3e, right panel).

File Name: Supplementary Data 4
Description: List of genes differentially expressed between non-HE cells and HE cells at E11 (Fig. 3f, left panel).

File Name: Supplementary Data 5
Description: List of genes differentially expressed between non-HE cells and HE cells at E10 (Fig. 3f, right panel).

File Name: Supplementary Data 6
Description: List of genes differentially expressed between E11 and E10 dorsal wIAHCs (Fig. 6b).

File Name: Supplementary Data 7
Description: List of genes differentially expressed between E11 and E10 ventral wIAHCs (Fig. 6c).

File Name: Supplementary Data 8
Description: List of genes differentially expressed between E11 ventral and dorsal wIAHCs (Fig. 6d).

File Name: Supplementary Data 9
Description: List of genes differentially expressed between E10 ventral and dorsal wIAHCs (Fig. 6g).
